# Migraine triggers, phases, and classification using machine learning models

**DOI:** 10.3389/fneur.2025.1555215

**Published:** 2025-05-09

**Authors:** Anusha Reddy, Ajit Reddy

**Affiliations:** ^1^San Juan Bautista School of Medicine, Caguas, Puerto Rico, United States; ^2^Independent Researcher, Monmouth County, NJ, United States

**Keywords:** migraine triggers, migraine phases, migraine types, logistic regression, support vector machine, random forest, neural networks

## Abstract

**Background:**

In many countries, patients with headache disorders such as migraine remain under-recognized and under-diagnosed. Patients affected by these disorders are often unaware of the seriousness of their conditions, as headaches are neither fatal nor contagious. In many cases, patients with migraine are often misdiagnosed as regular headaches.

**Methods:**

In this article, we present a study on migraine, covering known triggers, different phases, classification of migraine into different types based on clinical studies, and the use of various machine learning algorithms such as logistic regression (LR), support vector machine (SVM), random forest (RF), and artificial neural network (ANN) to learn and classify different migraine types. This study will only consider using these methods for diagnostic purposes. Models based on these algorithms are then trained using the dataset, which includes a compilation of the types of migraine experienced by various patients. These models are then used to classify the types of migraines, and the results are analyzed.

**Results:**

The results of the machine learning models trained on the dataset are verified for their performance. The results are further evaluated by selective sampling and tuning, and improved performance is observed. The precision and accuracy obtained by the support vector machine and artificial neural network are 91% compared to logistic regression (90%) and random forest (87%). These models are run with the dataset without optimal tuning across the entire dataset for different migraine types; which is further improved with selective sampling and optimal tuning. These results indicate that the discussed models are relatively good and can be used with high precision and accuracy for diagnosing different types of migraine.

**Conclusion:**

Our study presents a realistic assessment of promising models that are dependable in aiding physicians. The study shows the performance of various models based on the classification metrics computed for each model. It is evident from the results that the artificial neural network (ANN) performs better, irrespective of the sampling techniques used. With these machine learning models, types of migraines can be classified with high accuracy and reliability, enabling physicians to make timely clinical diagnoses of patients.

## Introduction

### Background

Migraine is a severe and disabling neurological condition, ranked by the World Health Organization as the sixth most disabling disorder globally and the most disabling of all neurological disorders. Unfortunately, this ranking continues to only increase with time. Migraine has a 1-year prevalence of 15–18% worldwide when both episodic and chronic forms are included and places a substantial financial burden on global economies. It predominantly affects women over men at a ratio of 3:1 and significantly impacts the quality of life, particularly during the peak years of productivity. Unilateral, throbbing head pain attacks, with sensitivity to movement, visual stimuli, auditory stimuli, and other afferent inputs characterize migraine. Other symptoms such as tiredness, irritability, reduced concentration, and yawning can precede the headache by up to 48 h in the premonitory phase. The majority of attacks are followed by hours to days of feeling unwell, usually with tiredness, called the postdrome phase. In addition, in approximately one-third of migraine patients, their attacks are associated with neurological deficits, which include cortical perturbations that are collectively referred to as migraine aura (1, 37). Despite awareness regarding migraine in developed countries, without exaggeration, it can be said that certain cases of migraine, such as sporadic hemiplegic migraine, which is estimated to affect 0.005% of the population, go undiagnosed even among the best of physicians. Migraine and related conditions are generally treated as a headache disorder, yet much is unknown about the public health impact of these conditions. Although the general understanding among the healthcare community concerning headache disorders is incomplete, our knowledge of healthcare resource allocation to headaches is scant; however, there is substantial evidence that very large numbers of individuals suffering from headaches do not receive effective healthcare. The issues related to this condition vary worldwide, but poor awareness in the context of limited resources in healthcare seems to be one of the many problems, and the collection and compilation of data in terms of quantity and quality is also scarce among many countries. In medical schools, there is limited teaching on the subject of headache disorders, with insufficient time allocated for studying headache disorders and training related to their management.

### Migraine

Migraine, for a long period of time, was often misjudged and treated as an illness among women who suffered migraine attacks as a result of stress. The majority of individuals are likely to experience a couple of migraine attacks during their lifetime. During the course of migraine attacks, very painful headaches, nausea, vomiting, sensitivity to light noise, and other external stimuli can be experienced. A patient is said to be suffering from chronic migraine if there are frequent attacks. A third of the patients with chronic migraine may also experience brain loss, resulting in the loss of part of one's vision, which can manifest as paralysis of one side. Research has also shown that repeated migraine attacks can cause brain damage (2, 3).

Migraine is a disorder that almost certainly has a genetic basis (2–4), but environmental factors play a significant role in how it affects those who have it. Recent research has shown the role of glial cells in migraine. Pathophysiologically, activation of a mechanism deep in the brain causes the release of pain-producing inflammatory substances around the nerves and blood vessels of the head. However, the reason behind the periodic occurrence of migraine attacks and the mechanisms that lead to the spontaneous resolution of these attacks remain uncertain (2, 36).

In many cases of migraine, the pain affects only half of the head. However, the pain is sometimes felt bilaterally at the back or front of the head and, rarely, over the body and face. The pain is typically throbbing and sometimes pulsatile in nature, typically increasing with any movement of the body or head (5).

Migraine is a common chronic headache disorder that is characterized by recurrent attacks that last from 4 to 72 h, with a pulsating quality. Migraine is the most common cause of headache, and its intensity is categorized as mild, moderate, and severe, with any routine physical activity aggravating it. It is attributed to meningeal perivascular pain fiber activation and increased sensitization of central pain neurons that process information from intracranial structures and extracranial skin and muscles (5, 6).

### Migraine—triggers

Several intrinsic or extrinsic factors can trigger a migraine attack. A migraine trigger is any environmental, dietary, or physiologic factor that can provoke migraine activity in the brain. It is very important to have sufficient information or knowledge about migraine triggers for the proper management of patients (6, 7).

- **Environmental triggers**—Odors such as perfume, chemicals, and petroleum products; bright lights; noise; and other excessive sensory stimuli can act as triggers. Painful stimuli that trigger migraines usually occur in the head and neck, and the most common are neck injury and spasms, temporomandibular joint pain, and sinus inflammation. Many migraine patients report that they are affected by weather changes.
- **Food triggers**—Byproducts of aging food are found in fermented products such as red wine, aged cheese, and yeast in fresh bread and yogurt, as well as coffee, chocolate, MSG, and the nitrates used as preservatives in many of our prepackaged foods.
- **Physiological triggers**—Stress, fatigue, lack of sleep, variations in sleep schedule, sleeping too much, hunger, exercise, pain, and hormone changes, such as a drop in estrogen levels before the menstrual period or after menopause.
- **Medication triggers**—Many medications can trigger migraines, including pain relievers, sleeping pills, and antidepressants. Treatment of chest pain or angina with nitrates is known to trigger migraines.

### Migraine—phases

Migraine is characterized by multiple phases: premonitory, aura, headache, postdrome, and interictal (8).

- **Premonitory phase—**The premonitory phase begins as early as 3 days before the headache phase and involves a complex interplay between various cortical and subcortical brain regions, including the hypothalamus and brainstem nuclei that modulate nociceptive signaling (8). Migraine attacks often come before premonitory symptoms. Possible premonitory symptoms included concentration problems, depression, food cravings, physical hyperactivity, irritability, nausea, phonophobia, fatigue, sleep problems, stressed feelings, stiff neck, and yawning (9, 10).
- **Aura phase—**In one-third of patients, an aura phase may occur during some attacks and likely correlates with a cortical spreading depression-like event: a slowly propagating wave of neuronal and glial cell depolarization and hyperpolarization (8, 10, 11).
- **Headache phase—**The headache phase involves activating the trigeminovascular system. The characteristic throbbing pain of migraine headaches is widely accepted to be the result of trigeminovascular pathway activation. The trigeminovascular pathway is well characterized, and its anatomy and physiology explain the distribution of the pain observed in migraine (8, 10, 38).
- **Postdrome phase—**The postdrome phase is the final stage of attack. Symptoms mimic the first stage and last from hours to days, only to disappear, leaving behind the feeling of hangover or tiredness (7). Non-headache symptoms may start before the headache or during the premonitory, headache, or postdrome phases. These symptoms involve brain activation of cortical and subcortical structures. Non-headache symptoms may persist for 1–2 days after the headache resolves in the postdrome or recovery phase (12). A higher proportion of individuals experience postdrome, during which they may experience a grumbling headache, a bruised feeling in the head, fatigue, and nausea, and a continuing sensitivity to lights, noises, smells, and movement (5, 8). This phase can be equally or more disabling than the preceding phases (10).
- **Interictal phase—**The interictal phase is the interval between two migraine attacks during which patients are symptom-free (8).

### Classification of migraine

Due to the lack of pathognomonic markers for migraine, co-occurrence of migraine subtypes and tension-type headaches within the same individual, and lack of validity of the inclusion criteria and boundaries between migraine and other headache subtypes, the classification of migraine has been delayed. There is an association between the subtypes of migraine, and its nature is defined by the International Headache Society Criteria (13).

**Migraine without aura**—Migraine without aura is a recurrent headache disorder in which attacks last anywhere from 4 to 72 h. It has some common symptoms, with the most common symptoms being unilateral location and a pulsating quality, which can range from moderate-to-severe intensity. The intensity of the headache may worsen with some routine physical activities and is associated with nausea and/or vomiting, photophobia, and phonophobia (7, 14).

**Migraine with aura—**Migraine with aura is observed with recurrent attacks, typically lasting only minutes and resolving completely afterward, with unilateral, fully reversible visual, sensory, or other central nervous system symptoms that usually develop gradually and are followed by headache and other associated migraine symptoms (7). The aura precedes before the headache starts, which is described as a complex of neurological symptoms. The symptoms may be visual or sensory and can include blind spots, zig-zag lines, shimmering stars, changes or loss in vision, and flashes of light (14, 15).

**Migraine with typical aura—**Migraine with typical aura is characterized by any of the following symptoms alone or in combination with visual, sensory, and speech/language symptoms, with no motor weakness. The gradual development of positive and negative features such as visual disturbances or numbness characterizes it. Each symptom lasts no longer than 1 h and fully resolves once the aura phase passes (complete reversibility) (7, 14).

- **Typical aura with headache:** Migraine with typical aura in which the aura is accompanied with or without migraine characteristics and followed by headache within 60 min (7, 14).
- **Typical aura without headache:** The aura that is neither accompanied nor followed by any headache (7, 14).

**Migraine with brainstem aura—**Migraine with brainstem aura has symptoms that originate from the brainstem and has no motor weakness. It was formerly known as a basilar-type aura. The aura can include vertigo, dizziness, and vision changes (1, 14, 15).

**Hemiplegic migraine—**This is a subtype of migraine with aura that includes motor weakness (1, 7, 11, 14).

- Familial hemiplegic migraine (FHM): This type of migraine affects individuals with at least one first- or second-degree relative with migraine aura including motor weakness (1, 7, 11, 14).
- Sporadic hemiplegic migraine (SHM): This type of migraine affects individuals with no first- or second-degree relative with migraine aura including motor weakness (14–17).

**Figure 1:**
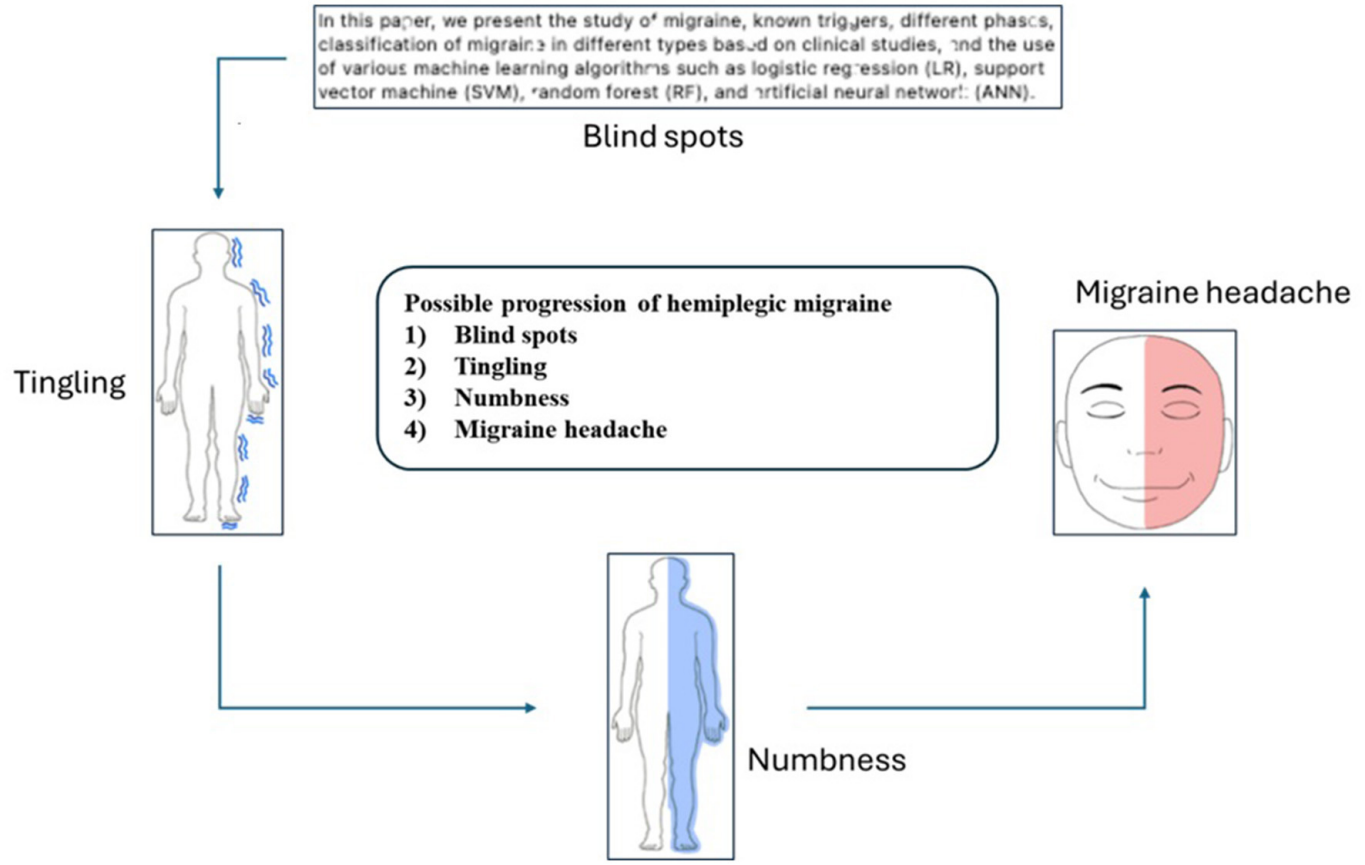
Possible progression of hemiplegic migraine.

Some patients experience difficulty reading the text during the initial stage of a hemiplegic migraine, as the characters appear illegible. This is followed by a tingling sensation and numbness, a precursor to the migraine headache shown in Figure 1.

**Retinal migraine—**This is a type of migraine in which the primary symptom is a fully reversible visual disturbance affecting only one eye (monocular). It is usually characterized by temporary blurring, shimmering, or loss of vision. The migraine typically resolves completely within a short period and is often accompanied by a headache (14).

**Chronic migraine—**A headache occurring for 15 days or more per month for more than 3 months and at least 8 days per month has the features of migraine headache (7, 14).

**Probable migraine—**Migraine-like attacks that are missing one of the required features to fully meet all criteria for a specific type or subtype of migraine, coded above and do not fulfill the criteria for another headache disorder (14).

- Probable migraine without aura.
- Probable migraine with aura.

**Benign paroxysmal vertigo—**It is characterized by a sudden, intense spinning sensation (vertigo) that lasts seconds to minutes and resolves spontaneously. Symptoms include dizziness, blurred vision, nausea, and vomiting (14, 18).

**Benign paroxysmal torticollis—**It is a rare, temporary condition that causes involuntary head and neck movements. It typically occurs in infants and young children, with an onset in the first year. It is accompanied by neck stiffness and may be vomiting (14).

**Diagnosing migraine—**The International Classification of Headache Disorders (ICHD) criteria for migraine and other primary headaches consistently include the phrase “not attributed to another disorder” and recommend that secondary headache disorders, indicated by the patient's history and/or physical and/or neurological examinations, be ruled out through ”appropriate investigations.” The presence of red flags, more so than yellow flags, raises the likelihood of a secondary cause of headache and should trigger further evaluation (14, 19).

Some headaches may begin and end with sleep without warning signs. In other instances, the headache may be preceded by a prodromal phase that exhibits symptoms such as extreme fatigue, depression, irritability, food cravings, a sensation of intense excitement or happiness, constipation, neck stiffness, increased yawning, abnormal sensitivity to light, sound, and smell, and immediately before the headache phase, an aura phase occurs, featuring a variety of focal cortically mediated neurological symptoms (11). Premonitory symptoms indicating an impending migraine headache have been recognized for many years. Even after the headache subsides, many patients experience a postdrome phase that lingers for 1–2 days (20). The range of typical symptoms that patients with migraine encounter is reflected in the ICHD criteria for diagnosing a migraine.

### Rationale for machine learning techniques

Previous studies using machine learning techniques using similar models to diagnose migraines using support vector machine, random forest, and artificial neural networks (21–24) have yielded promising results regarding prediction accuracy. Recent studies have strongly proposed using machine learning models, the large language model (LLM) to diagnose headache disorders, and the digital twin for the human body (25, 26). However, it is unclear from these studies if the large language models are substantially better for smaller datasets, given the complexity, accuracy, and precision obtained. Machine learning (ML) models are discussed for the prediction of anti-calcitonin gene-related peptide (CGRP) response in the treatment of patients with migraine (27). Further studies using machine learning (ML) and deep learning (DL) are discussed (28) for evaluating datasets, including brain MRIs of patients with migraine and post-traumatic headaches.

The real question that we attempt to address is that, although migraine is a debilitating condition experienced by many throughout the world, diagnosing and treating this condition is not always done correctly by many physicians. Patients often go years without a correct diagnosis and often undergo unnecessary imaging and tests to diagnose their condition. With the machine learning techniques discussed in the study, diagnosing migraines can be more efficient without requiring elaborate diagnostic tests.

### Objectives of the methodology

Our objective in this study is to provide a framework, ensure the accuracy and reliability of results by minimizing bias, enable the generalization of findings to a broader population, and ultimately provide insights and understanding into the topic being investigated by utilizing appropriate methods to diagnose the types of migraines that individuals with migraine experience. As part of developing the necessary diagnostic tool, we decided to use machine learning models as diagnostic capabilities to aid physicians in undecided cases in better understanding migraine and helping the patient with the correct diagnoses and treatment.

## Methods

### Study design

We present a list of classification algorithms with an introduction to their fundamental concepts. These algorithms are used in models to classify different types of migraine.

### Logistic regression (LR)

Logistic regression is a classification model that performs particularly well on linearly separable classes. It is one of the most widely used algorithms for classification. The idea behind logistic regression as a probabilistic model can be explained by introducing the odds ratio, which represents the odds in favor of a particular event. The odds ratio can be written as $\frac{1}{\left(1-p\right)}$, where *p* stands for the probability of the positive event. The term “positive event” does not necessarily mean good but refers to the event we want to predict. The logit function can be defined as the logarithm of the odds ratio:


$$\begin{array}{l} logit\left(p\right)=\log\frac{1}{\left(1-p\right)} \end{array}$$

The logit function takes input values in the range 0–1 and transforms them to values over the entire real number range, which we can use to express a linear relationship between feature values and the log odds:


$$\begin{array}{l} logit\left(p\left(y=1\;|\;x\right)\right)=\;\sum_{k=0}^{n}w_{k}x_{k}=\;{\text{w}}^{\text{T}}\text{x}, \end{array}$$

where (*p*(*y* = 1 | *x*) is the conditional probability that a particular sample belongs to class 1, given its features *x*. We are particularly interested in predicting the likelihood that a certain sample belongs to a particular class, which is the inverse form of the logit function. It is also called the logistic function, which is sometimes abbreviated as the sigmoid function due to its characteristic S shape.


$$\begin{array}{l} \emptyset \left(z\right)=\frac{1}{1-z^{-\;1}}, \end{array}$$

where *z* is the net input, that is, the linear combination of weights and sample features, and can be calculated as *z* = **w**^**T**^**x** (29).

### Support vector machine (SVM)

Support vector machine is a powerful and widely used learning algorithm. It can perform both classification and regression tasks. In SVMs, the optimization objective is to maximize the margin. The margin is defined as the distance between the separating hyperplane (decision boundary) and the training samples that are closest to this hyperplane, which are the support vectors. The rationale behind having decision boundaries with large margins is that they tend to have a lower generalization error, whereas models with small margins are more prone to overfitting. Consider positive and negative hyperplanes parallel to the decision boundary, which can be expressed as follows:


(1) $$w_{0}+\;w^{T}x_{pos}=1\;$$

(2) $$w_{0}+\;w^{T}x_{neg}=-1$$

Subtracting (2) from (1), we get


$$w^{T}\left(x_{pos}-x_{neg}\right)=2\;$$

We can rewrite the equation as follows:


$$\frac{w^{T}\left(x_{pos}-x_{neg}\right)}{\left\|w\right\|}=\frac{2}{\left\|w\right\|},$$

where ||**w**|| is the normalizing function, and the left side of the preceding equation can be interpreted as the distance between the positive and negative hyperplanes, which is the margin we want to maximize (30).

In solving non-linear problems, kernel methods deal with linearly inseparable data by creating non-linear combinations of the original features and projecting them onto a higher-dimensional space via a mapping function ϕ(·), where they become linearly separable. One of the most widely used kernels is the radial basis function kernel (RBF kernel) (30) or Gaussian kernel.

### Random forest (RF)

The random forest algorithm is a powerful tree-learning method that creates several decision trees during training. Each tree is constructed using a random subset of the dataset to measure a random subset of features in each partition. This randomness introduces variability among individual trees, reducing the risk of overfitting and improving overall prediction performance. Random forest leverages the power of the ensemble technique by constructing a group of decision trees. Random forest employs random feature selection, and a random subset of features is chosen during the training of each tree. This randomness ensures that each tree focuses on different aspects of the data, fostering a diverse set of predictors within the ensemble. The bootstrap aggregation or bagging technique is the main strategy of random forest's training strategy, which involves creating multiple bootstrap samples from the original dataset and allowing instances to be sampled with replacement. This results in different subsets of data for each decision tree, introducing variability in the training process and making the model more robust. Each decision tree makes predictions in the random forest by casting its vote for classification tasks or by averaging in the case of regression tasks, and the final prediction is determined by the most frequent prediction across all trees. This collaborative decision-making process, supported by multiple tree insights, provides an example of stable and precise results (31, 32).

### Artificial neural network (ANN)

**Figure 2:**
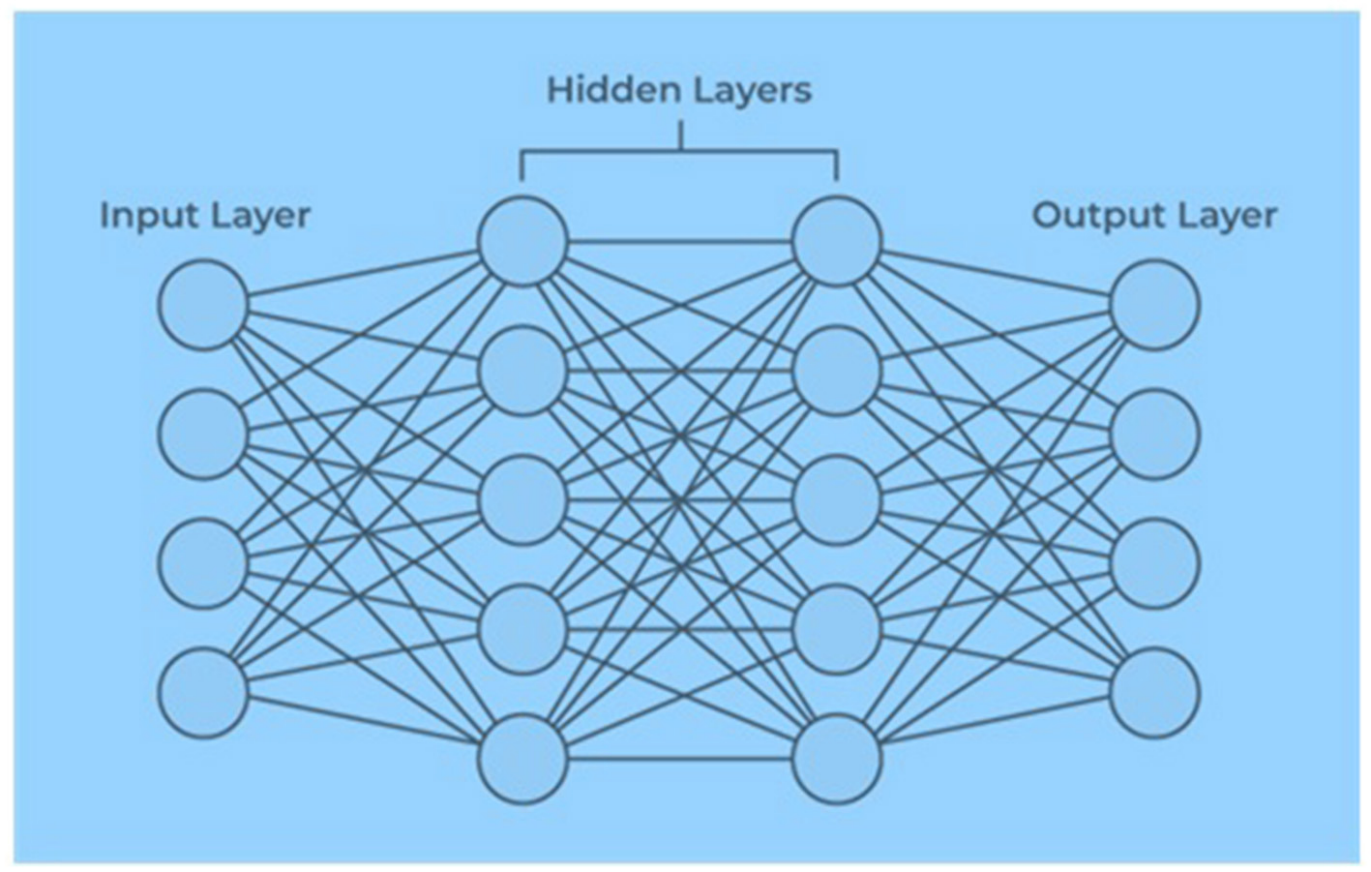
Layers of artificial neural network.

An artificial neural network contains artificial neurons arranged in layers constituting a system's whole artificial neural network. Depending on the complexity of the problem, each layer can contain anywhere from a dozen neurons to millions of neurons, as this determines the complex neural networks required to learn the hidden patterns in the dataset. Artificial neural networks have input, output, and hidden layers, as shown in Figure 2.

The input layer receives data from the outside world, which the artificial neural network needs to analyze or learn about. Then, these data pass through one or multiple hidden layers that transform the input into valuable data for the output layer. Finally, the output layer provides an output in the form of a response of the artificial neural network to the input data provided. In the majority of neural networks, units are interconnected from one layer to another. Each of these connections has weights that determine the influence of one neuron on another. As the data transfer from one neuron to another, the neural network learns more and more about the data, eventually resulting in an output from the output layer (21). The input vector is presented to the input layer, and each input neuron's production equals the corresponding component in the vector. Each hidden neuron is the weighted sum of its inputs denoted as follows:


$$\begin{array}{l} net_{j}=\sum_{i=0}^{d}x_{i}w_{ji}=\;{\text{w}}_{j}^{t}\text{x}, \end{array}$$

where *i* indexes neurons in the input layer, *j* indexes the neurons in the hidden layer, and *w*_*ji*_ denotes the input to hidden layer weights at the hidden neuron *j*. Each hidden neuron emits an output that is a non-linear function, which is *y*_*j*_ = *f*(*net*_*j*_) and


$$f\left(net\right)=Sgn\left(net\right)\equiv \{\begin{array}{c} \;1\;if\;net\;\geq 0\\ -1\;if\;net<0 \end{array}$$

Each output neuron computes its net activation based on the hidden neuron signals as follows:


$$\begin{array}{l} net_{k}=\sum_{i=0}^{n_{H}}y_{j}w_{kj}=\;{\text{w}}_{k}^{t}\text{y} \end{array}$$

where *k* indexes neurons in the output layer, *n*_*H*_*denotes the number of hidden neurons*, and the output neuron computes the non-linear function of its *net*, thereby emitting,


$$\begin{array}{l} z_{k}=f\left(net_{k}\right) \end{array}$$

### Dataset collection and compilation

The dataset used for training the above-discussed models consists of 400 medical records of users diagnosed with various pathologies associated with migraines. During the first quarter of 2013, trained medical personnel at the Centro Materno Infantil de Soledad, consisting of medical staff and physicians, collected data on patients with different types of migraines and compiled it with the required attributes and descriptions (21, 33), as shown in Table 1. The dataset used is based on the retrospective study. A prospective study of individuals with migraine is being conducted, which has not been made available for training the machine learning models.

**Table 1:** Data on patients with migraine collected by medical personnel at the Centro Materno Infantil de Soledad.

**Attribute**	**Description**
1) Age	Patient's age
2) Duration	duration of symptoms in the last episode in days
3) Frequency	Frequency of episodes per month
4) Location	Unilateral or bilateral pain location (None - 0, Unilateral - 1, Bilateral - 2)
5) Character	Throbbing or constant pain (None - 0, Throbbing - 1, Constant - 2)
6) Intensity	Pain intensity, i.e., mild, medium, or severe (None - 0, Mild - 1, Medium - 2, Severe - 3)
7) Nausea	Nauseous feeling (Not - 0, Yes - 1)
8) Vomit	Vomiting (Not - 0, Yes - 1)
9) Phonophobia	Noise sensitivity (Not - 0, Yes - 1)
10) Photophobia	Light sensitivity (Not - 0, Yes - 1)
11) Visual	Number of reversible visual symptoms
12) Sensory	Number of reversible sensory symptoms
13) Dysphasia	Lack of speech coordination (Not - 0, Yes - 1)
14) Dysarthria	Disarticulated sounds and words (Not - 0, Yes - 1)
15) Vertigo	Dizziness (Not - 0, Yes - 1)
16) Tinnitus	Ringing in the ears (Not - 0, Yes - 1)
17) Hypoacusis	Hearing loss (Not - 0, Yes - 1)
18) Diplopia	Double vision (Not - 0, Yes - 1)
19) Visual defect	Simultaneous frontal eye field and nasal field defect in both eyes (Not - 0, Yes - 1)
20) Ataxia	Lack of muscle control (Not - 0, Yes - 1)
21) Conscience	Jeopardized conscience (Not - 0, Yes - 1)
21) Paresthesia	Simultaneous bilateral paresthesia (Not - 0, Yes - 1)
23) DPF	Family background (Not - 0, Yes - 1)
24) Type	Diagnosis of migraine type:
	1) Typical aura with migraine
	2) Migraine without aura
	3) Typical aura without migraine
	4) Familial hemiplegic migraine
	5) Sporadic hemiplegic migraine
	6) Basilar-type aura
	7) Other

The physician made the diagnosis, and the attribute “Type” indicates the diagnosis of the type of migraine that is determined by the consulting physician based on the symptoms and medical history of the patient, arriving at one of the following classifications:

1. Typical aura with migraine.
2. Migraine without aura.
3. Typical aura without migraine.
4. Familial hemiplegic migraine.
5. Sporadic hemiplegic migraine.
6. Brainstem aura (basilar-type aura).
7. Other.

### Data analysis

**Figure 3:**
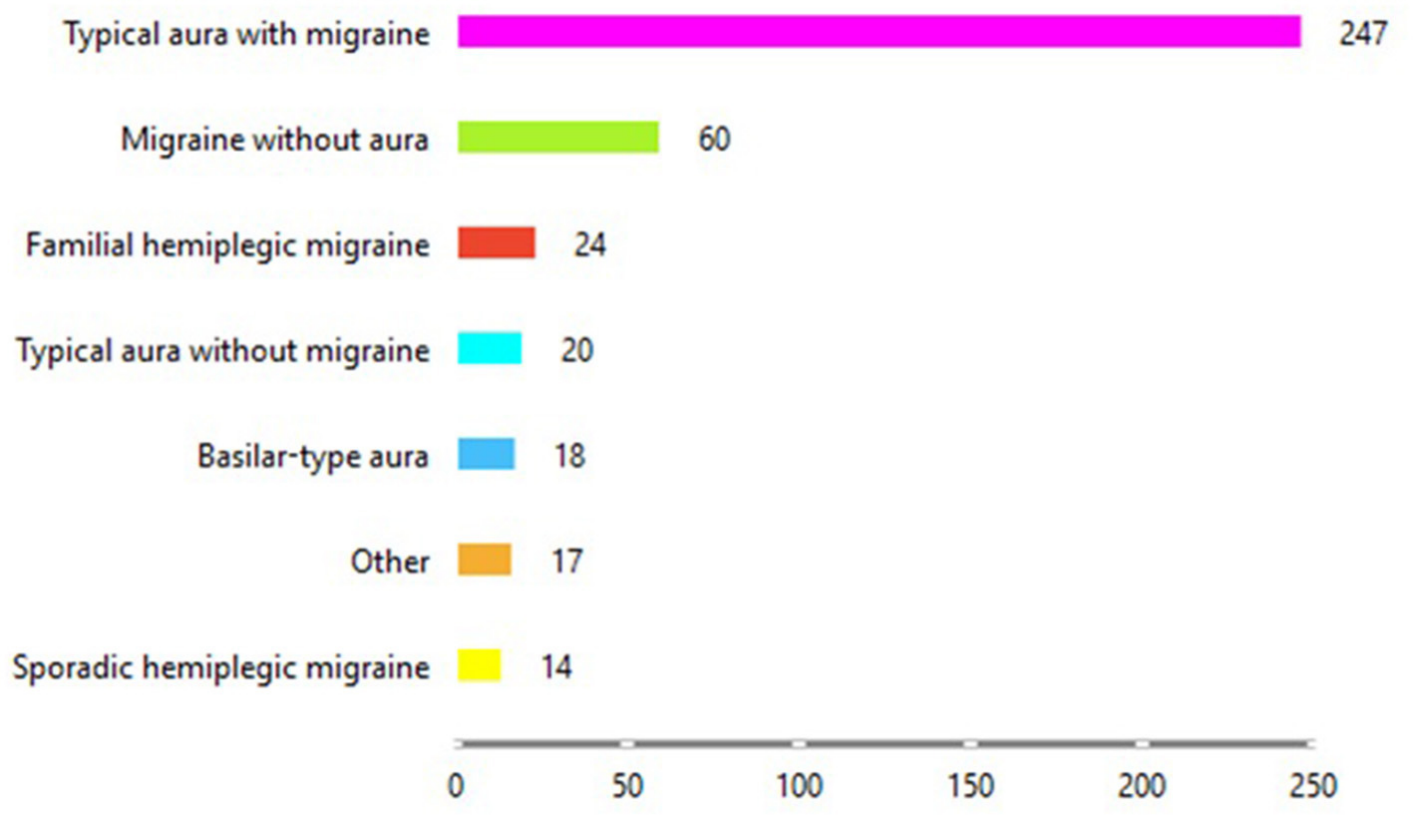
Distribution of migraine types in the dataset.

Figure 3 shows the distribution of the type of migraine each patient is diagnosed with at a given age from the dataset. It is clear from Figure 3 that most of the patients have been diagnosed with “Typical aura with migraine,” followed by patients with “Migraine without aura.” The group with the fewest patients is those with “Sporadic hemiplegic migraine.”

### Diagnostic methods and classification methodology

The diagnostic methods (34, 35) discussed combine many elements of artificial intelligence (AI), machine learning (ML), and statistics. The methods and classification models discussed in the section study design, namely, logistic regression (LR), support vector machine (SVM), random forest (RF), and artificial neural network (ANN), are used to classify the type of migraine from the information collected in the dataset. The dataset consists of 24 variables, split as 80% into training data and 20% into testing data. The metrics obtained with each of the models are compared.

**Figure 4:**
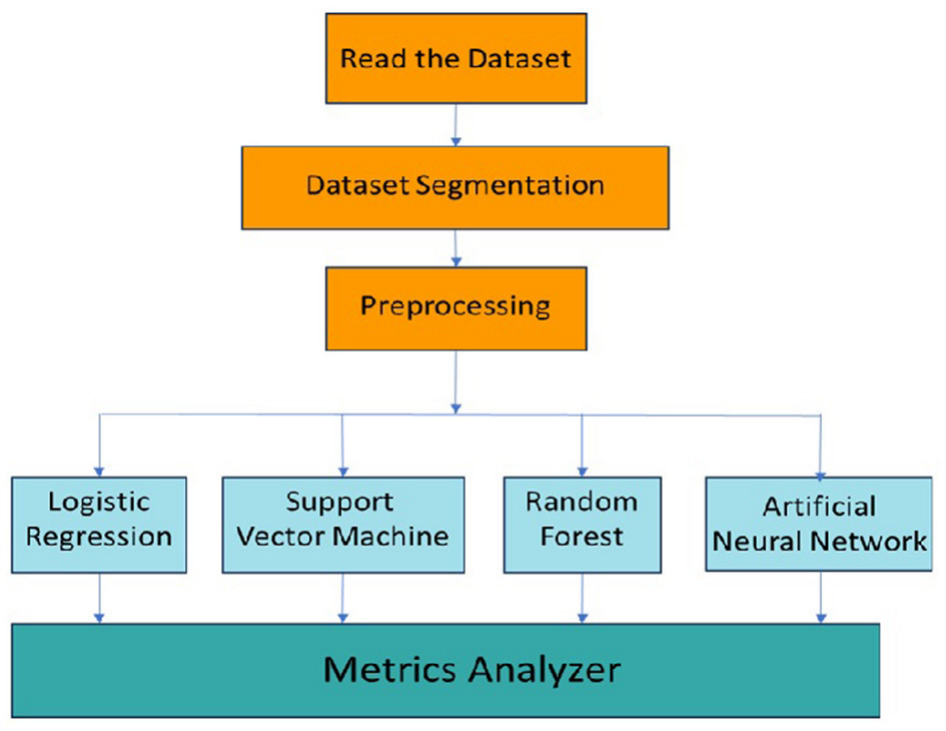
Migraine classification methodology.

The machine learning methodology for the classification of migraine is shown in Figure 4. The dataset is read from a file and stored in a data table. The dataset is then segmented into training and testing data, which is preprocessed before input into the respective machine learning models for training and testing. The outputs of the respective models are then analyzed for various classification metrics based on which the performance and suitability of each model are determined.

### Classification metrics

- **The area under ROC curve (AUC)—**The area under the ROC curve (AUC) represents the probability that the model will rank the positive higher than the negative if given a randomly chosen positive instance and a randomly chosen negative instance.
- **Classification accuracy—**The classification accuracy is the ratio of correctly predicted instances to the total number of instances in the dataset. It is useful when the class distribution is balanced.

- **Precision—**Precision refers to the proportion of accurate positive predictions among all positive predictions, which indicates that it measures how accurate the positive predictions are.
- **Recall—**Recall is the proportion of true positive predictions among all actual positive instances. It measures the classifier's ability to identify positive instances correctly.
- **Specificity—**Specificity refers to the model's capability to correctly identify negative cases.

## Results

The dataset comprises 400 patients, based on the retrospective medical records of patients with various types of migraine. The compiled dataset presented in Table 1 with the various attributes is used. The dataset is read from the database, segmented into training and testing data, and preprocessed, wherein the data are checked for noise, errors, and other anomalies. The preprocessed data are then used as input to the various models, as shown in Figure 4. Initially, with random sampling, we run the models with the dataset, train the models, and compute the classification metrics given in Table 2. In the random sampling method, we adopt stratified sampling, where the samples of the patient's data are divided into subsets called strata from which samples are drawn. It can be observed that, with the random sampling strategy, with no optimal tuning, the artificial neural network and support vector machine appear to perform well with ~91% precision and accuracy as compared to the other models, such as logistic regression (90%) and random forest (87%). The model is then run with selective sampling, a non-probabilistic sampling technique, and tuning for the given dataset wherein the sample is picked up from a larger sample size based on the assessment, and then the metrics are computed as shown in Table 3. The results indicating that we can obtain high accuracy and precision are further discussed. The order of usage of these for the given dataset and sampling strategy is as follows: artificial neural network (99%), random forest (98%), support vector machine (96%), and logistic regression (95%). These results also indicate that the accuracy and precision of these models can vary for the same dataset based on the sampling and the estimators used. For the artificial neural network, we have used Adam as the solver and Relu as the activation; for the case of random forest, we have used a minimal number of trees with growth control; and for the support vector machine, we have used a linear kernel and ridge (L2) regularization type for logistic regression.

**Table 2:** Metrics with random sampling.

**Model**	**AUC**	**CA**	**Precision**	**Recall**	**Specificity**
Logistic regression	0.973	0.901	0.896	0.901	0.911
Random forest	0.968	0.870	0.846	0.870	0.893
Support vector machine	0.973	0.917	0.912	0.917	0.929
Artificial neural network	0.972	0.914	0.910	0.914	0.936

**Table 3:** Metrics with selective sampling.

**Model**	**AUC**	**CA**	**Precision**	**Recall**	**Specificity**
Logistic regression	0.990	0.958	0.958	0.958	0.950
Random forest	0.998	0.980	0.980	0.980	0.975
Support vector machine	0.988	0.965	0.965	0.965	0.951
Artificial neural network	1.000	0.995	0.995	0.995	1.000

## Discussion

With tuning, it can be observed that all the models perform better and that the precision and accuracy of random forest (98%) and artificial neural network (99%) are high. However, in both cases, the artificial neural network performs better, is self-adaptive, and does not require prior information with regard to the type of migraine being classified. In addition, it is important to note that the artificial neural network can learn complex behaviors from the dataset and infer correctly. The results are similar to those obtained in other studies (21–23). In this study, we initially evaluated several machine learning models and selected only those that are suitable for the dataset and the topic. For smaller datasets, both random forest and support vector machine perform reasonably well. We have further tested this model with additional test data, and the results comply with our study. Further research is required with not only different datasets but also very large datasets and the use of artificial neural networks, deep neural networks (DNNs), and large language models (LLMs) with these datasets. Additional attributes currently not considered in this study could be due to overfitting, where the model memorizes the training data instead of learning generalizable patterns, which is a significant challenge and perform poorly in real-world scenarios. The methods discussed and validated suggest that they promise to reliably diagnose different types of migraine.

## Conclusion

In this study, we investigated the optimal choice of classifier for migraine detection. We present classification models based on the performance of various models in classifying migraine types. All of the models are evaluated based on their performance with regard to the classification metrics. Compared to all the models, the artificial neural network appears highly precise and accurate with random and selective sampling and tuning. Support vector machine (SVM) and random forest (RF) models performed efficiently and could be used as alternative models. Logistic regression for the given dataset did not perform well compared to the other models. With this finding, we conclude that the artificial neural network (ANN) is a dependable model that can aid a physician in diagnosing the type of migraine with certainty and in a reliable manner, and the significance of our study is related to this fact. Furthermore, the accuracy of these models could be increased by adding more patients with migraines, thereby increasing the dataset size to train these models. Future studies should involve obtaining a large dataset of individuals with migraine and using deep neural networks (DNNs) and large language models (LLMs).

## Clinical implications

- The classification methods discussed and validated in this study show promise for reliably diagnosing different types of migraine.
- These diagnostic methods could aid physicians' ability to accurately diagnose and effectively treat patients.

## Data Availability

The original contributions presented in the study are included in the article/Supplementary material, further inquiries can be directed to the corresponding author.
